# Editorial Change

**Published:** 1848-11

**Authors:** 


### EDITORIAL CHANGE.

Professor Lee, the learned and industrious editor of the New York Journal of Medicine, has retired from its management, and is succeeded by Dr. William Purple. Dr. Purple has for some time past assisted Dr. Lee in his editorial duties, and therefore enters upon his task ready harnessed for the work. While we congratulate Dr. Lee on his escape from the tripod, we sympathize with his successor in his mishap—for so we fear he will find his new *honor*, as he now probably deems it, but his *great trouble*, as we ween it will turn out. But notwithstanding our doubts, he has our best wishes and hearty welcome.

